# Ad-hoc preoperative management and respiratory events in pediatric anesthesia during the first COVID-19 lockdown–an observational cohort study

**DOI:** 10.1371/journal.pone.0273353

**Published:** 2022-08-18

**Authors:** Markus Zadrazil, Peter Marhofer, Werner Schmid, Melanie Marhofer, Philipp Opfermann

**Affiliations:** 1 Department of Anesthesia, General Intensive Care Medicine and Pain Therapy, Medical University of Vienna, Vienna, Austria; 2 Department of Anesthesia and Intensive Care Medicine, Orthopedic Hospital Vienna, Vienna, Austria; 3 Department of Special Anesthesia and Pain Medicine, Medical University of Vienna, Vienna, Austria; 4 Medical Student, Medical University of Innsbruck, Innsbruck, Austria; Kaohsuing Medical University Hospital, TAIWAN

## Abstract

**Background:**

Early pre-anesthetic management for surgery is aimed at identifying risk factors, which notably in children are mostly airway related. The first COVID-19 lockdown opened a unique ‘window of opportunity’ to study what impact an ad-hoc management strategy would bring to bear on intraoperative respiratory events.

**Methods:**

In this observational cohort study we included all patients with an American Society of Anesthesiology (ASA) Physical Status of I or II, aged 0 to ≤18 years, who underwent elective surgery at our center during the first national COVID-19 lockdown (March 15^th^ to May 31^st^, 2020) and all analogue cases during the same calendar period of 2017−2019. The primary outcome parameter was a drop in peripheral oxygen saturation (SpO_2_) below 90% during anesthesia management. The study is completed and registered with the German Clinical Trials Register, DRKS00024128.

**Results:**

Given 125 of 796 evaluable cases during the early 2020 lockdown, significant differences over the years did not emerge for the primary outcome or event counts (p>0.05). Events were exceedingly rare even under general anesthesia (n = 3) and non-existent under regional anesthesia (apart from block failures: n = 4). Regression analysis for SpO_2_ events <90% yielded no significant difference for ad-hoc *vs* standard preoperative management (p = 0.367) but more events based on younger patients (p = 0.007), endotracheal intubation (p = 0.007), and bronchopulmonary procedures (p = 0.001).

**Conclusions:**

Early assessment may not add to the safety of pediatric anesthesia. As a potential caveat for other centers, the high rate of anesthesia without airway manipulation at our center may contribute to our low rate of respiratory events.

## Introduction

Coronavirus disease 2019 (COVID-19) was declared a pandemic by the World Health Organization (WHO) on March 11^th^, 2020 [1]. Since its outbreak, many healthcare providers have been forced to modify their standard procedures to meet the special challenges arising from the crisis [2–9]. Elective surgery was postponed extensively and treatment of outpatients dramatically reduced [10, 11]. The rationale of subjecting clinical routines to these modifications was to ensure ‘social distancing’ aimed at minimizing the risk of transmitting COVID-19 between patients and medical staff [12].

To maintain a basic level of care in this situation, modifications to the daily clinical routines were implemented around the globe [5, 8, 13], including at the Medical University of Vienna in general and at our department (Anesthesia, General Intensive Care, and Pain Therapy) in particular. One of the domains affected was the standard process of obtaining informed consent in American Society of Anesthesiology Physical Status (ASA-PS, hereinafter simply ASA) I and II patients before anesthesia and surgery. While face-to-face contact between anesthetists and patients, under normal circumstances, takes place no later than the day before surgery [14, 15], no such contact was allowed under conditions of social distancing until immediately before surgery.

For some time, this was the only way to perform any elective surgery, including in pediatric cases with their even more complex process of obtaining informed consent, given the mandatory involvement of a parent or legal guardian. Controversial debates revolve around how extensive the process of preoperative evaluation and consent should be in anesthesia, how useful it is, and when it should be performed [16]. Medico-legal considerations have been driving an increasingly complex process of preoperative evaluation and patient information [15, 17]. From a strictly medical point of view, legal requirements aside, the point of preoperative evaluation in pediatric anesthesia is to ensure timely assessment and anticipation of potential risks related to anesthesia.

In children specifically, the preoperative assessment is executed to detect cardiac issues, undiagnosed syndromes, myopathies, mitochondrial disorders, innate metabolic disorders and potential airway-related problems [18] among others, whereas the latter is the main cause for perioperative complications in pediatric anesthesia [19]. When the first lockdown associated with the COVID-19 pandemic was imposed in early 2020, this opened a unique ‘window of opportunity’ to study if an ad-hoc approach to preoperative evaluation and obtaining informed consent would impact the frequency of intraoperative respiratory-related severe critical events (the most frequent complications in pediatric anesthesia in all age groups [19]) and of other anesthesia-related adverse events.

Our underlying hypothesis was that a streamlined protocol of ad-hoc management, as temporarily in effect at our center during the first COVID-19 lockdown (March 15 to May 31, 2020) would not have made a difference to the incidence of anesthesia-related events. As *primary outcome*, we defined events of peripheral oxygen saturation (SpO_2_) dropping below < 90% throughout anesthesia, both overall and stratified by age groups (months: 0−3, 4−12; years: 1−4; 5−12; 13−18). *Secondary outcomes* included the lowest SpO_2_ value, the lowest heart rate, time under anesthesia (defined as pulse oximeter time in the operating theater), time spent in the recovery room, unplanned intermediate care admissions, and anesthesia-related events of any kind (S1 Table). To verify our hypothesis, we designed an observational study of surrogate respiratory events, based on perioperative oxygen saturation (SpO_2_) and heart rate, in ASA I/II children and adolescents who underwent elective surgical procedures either during this period or, by comparison, during the same calendar period of the preceding three years.

## Materials and methods

### Study preparations and eligible patients

This observational study was approved by the institutional review board (ethics commission; re 1058/2021) on March 2^nd^, 2021, and entered into the DRKS trial register (DRKS 00024128) on March 4^th^, 2021. Considered for inclusion were all ASA I/II patients aged 0 and ≤ 18 years who underwent elective surgical procedures during the first COVID-19 lockdown in Austria, effective as of March 15^th^ through May 31^st^, 2020. In addition, patients meeting the same criteria were eligible who had been treated during the same calendar period of 2017 through 2019. Excluded were patients with an ASA status of III to V and/or patients undergoing emergency surgery. Collected data were retrospectively analyzed and validated in accordance with the STROBE statement for observational studies [20]. The STROBE checklist is attached in S2 Table.

### Standard versus ad-hoc management

Our standard process of preoperative evaluation and obtaining informed consent is normally scheduled to take place no later than 24 hours before surgery. It features a detailed dialogic conversation with a parent or legal guardian, including age-adjusted involvement of the child, and a brief physical examination as by stethoscope. For the ad-hoc process under the first lockdown, this standard procedure was modified such that physical contact and patient evaluation did not occur until the immediate preoperative phase in ASA I/II patients. Previous communications were handled by phone, including information on fasting requirements (six hours for solids, four hours for breast and formula milk, one hour for clear fluids) or cancellation of surgery (notably due to respiratory tract infection). The phone call was normally scheduled to take place no later than 24 hours before surgery and was executed by one member of the team of the department of pediatric anesthesia. If the parents/legal guardians mentioned a recent (within 14 days) manifest upper respiratory tract infection (URTI) the operation was postponed. If the parents/legal guardians mentioned a running nose or mild coughing the patient was not cancelled automatically, but was re-evaluated on the face-to-face meeting prior to the operation. This first physical contact with patients and parents/legal guardians, including signing of the informed consent form, took place about 30 minutes before anesthesia induction. Patients with mild URTI were not cancelled. Children with manifest respiratory problems were cancelled as according to our departmental standards. This “cancellation policy” has not been modified between the non-COVID era and the COVID era.

### Extraction and categorization of data

The baseline period was the first COVID-19 lockdown in Austria from March 15^th^ to May 31^st^, 2020. The periods for comparison accordingly were March 15^th^ to May 31^st^ of the previous three years 2017 through 2019. Data of ASA I/II children who underwent elective surgery during these periods were retrieved by systematic interrogation from the patient documentation and information system (PDMS, Philips Healthcare, Vienna, Austria) at the University Hospital Vienna. This system operates in real time and is managed for quality by data being exported and validated on a regular basis. For each patient, a specified dataset was extracted and categorized for baseline characteristics, type of anesthesia, perioperative data, intraoperative vital signs, and occurrence of complications.

### Screening and validation of data

Two observers (MM and PO) independently validated each case to rule out artefacts and verify that adverse events had actually occurred. Use of alternative data sources was made whenever needed, medical patient and discharge reports being two examples. Data were screened for completeness, consistency, and outliers before analysis. Any outliers in heart rate or SpO_2_ values were double-checked for plausibility. Wherever values were missing, alternative data sources such as hospital records were scrutinized before considering imputation. If required, missing values were replaced by appropriate subgroup medians.

### Study hypothesis

We hypothesized that the incidence of anesthesia-related adverse events in children and adolescents subjected to ad-hoc preoperative management during the Austrian lockdown period in early 2020 was equal to the incidence of events on record for the same kind of patients who had been managed by the standard preoperative approach during the same calendar period of the previous three years 2017 through 2019.

### Logistic regressions for surrogate events

The basic model analyzed whether the lockdown-specific approach of ad-hoc preoperative management was *per se* associated with a higher likelihood of such events. In the multivariate regression part, suspected confounders or effect modifiers were included based on established relationships between the explanatory (ad-hoc *vs* standard preoperative management) and the outcome (SpO_2_ events < 90%) variable, biological plausibility, and inhomogeneous distribution (p < 0.1), using a stepwise inclusion strategy to evaluate how these factors affected the explanatory variable.

### Statistical analysis

Patient characteristics are summarily reported as median values with interquartile ranges (IQR) or absolute numbers with percentages. All statistical comparisons were based on non-parametric Kruskal-Wallis tests for continuous variables and Pearson’s chi-squared tests for proportions. Comparisons of the primary endpoint were additionally stratified by age (see ‘Outcome parameters’ above), and the multiple regressions were based on a dichotomous (yes/no) endpoint of SpO_2_ events < 90% during anesthesia. Suspected confounders/effect modifiers were considered as co-variables in the multiple regression model, testing any metric co-variables for co-linearity before inclusion. Given a very low expected incidence of adverse events within the observation period [19], our statistical analysis included a multiple logistic regression for events of SpO_2_ falling below 90%, on the rationale that any drop in peripheral oxygen saturation below this threshold can be construed as a respiratory problem during anesthesia management. The limit of 90% of SpO_2_ was chosen as it represents a “margin of action” for the anesthesiologists in terms of bag ventilation, enhanced oxygen supply, further muscle relaxation amongst others. Data from regression are reported as raw and confounder-adjusted odds ratios with 95% confidence intervals. All tests were two-sided and differences considered significant at p < 0.05. Statistical software was used throughout (SPSS Statistics, version 24.0.0.0; IBM, Armonk, NY, USA). This study is registered with the German Clinical Trials Register, DRKS00024128.

## Results

**Fig 1:**
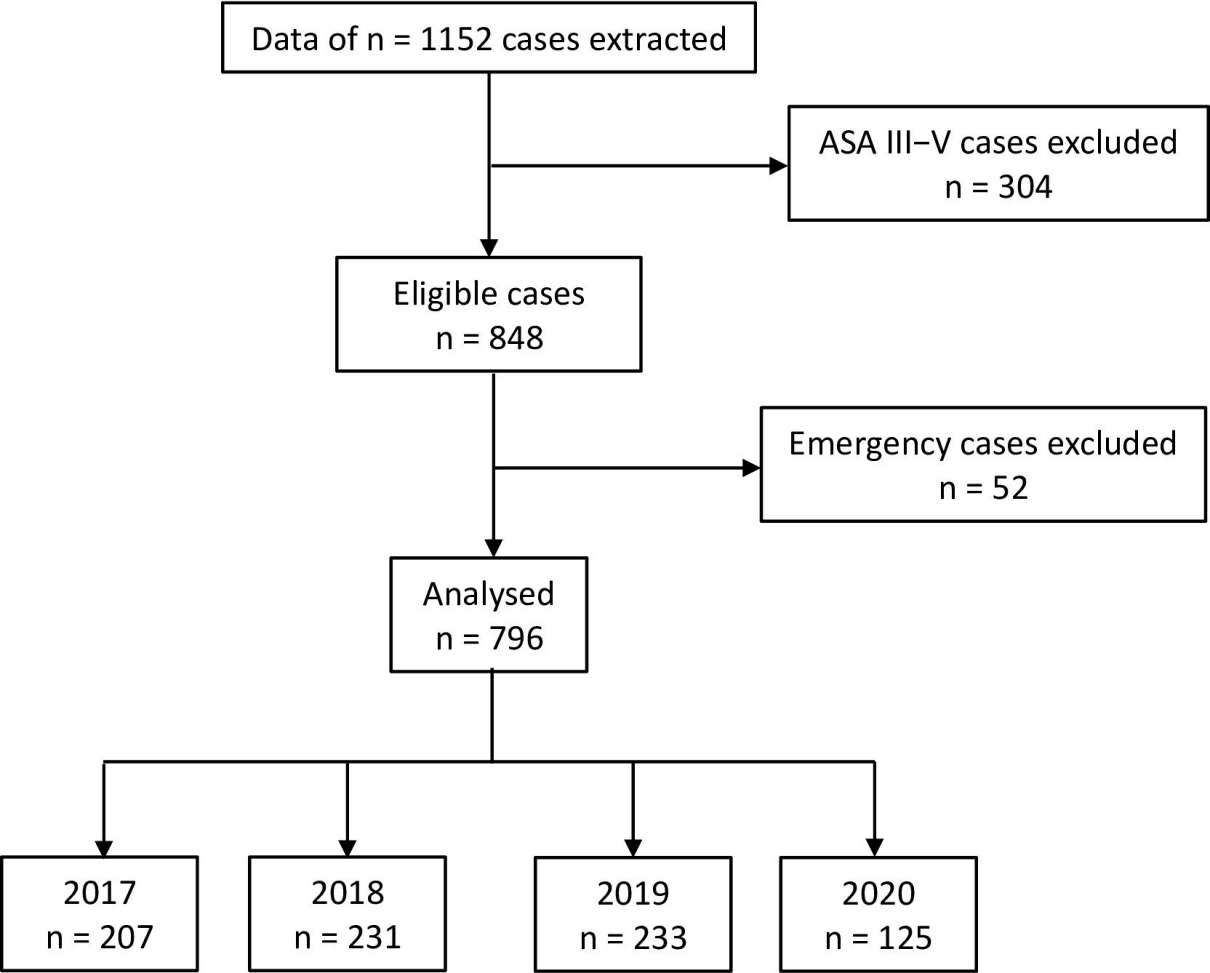
Study flow diagram according to STROBE.

In accordance with the STROBE statement Fig 1 illustrates the roadmap towards evaluable cases and how these fell into the four years of interest, 2020 indicating the time of the year when ad-hoc preoperative management was performed during the first COVID-19 lockdown, 2017 through 2019 the same calendar period of the preceding years. After exclusion of ASA III/IV/V cases (n = 304) and of urgent or emergency cases (n = 52), the underlying total population of 1152 was reduced to 796 evaluable cases. Of these, 125 had been anaesthetized under conditions of ad-hoc preoperative management in 2020, which represents a drop of elective surgical procedures in ASA I/II children and adolescents by 45.9% compared to the median case load over the same calendar period of the previous three years. In four cases we had missing values for our primary outcome parameter (SpO_2_) and the minimal heart rate due to electronically problems. In these cases, however, in the medical records an unremarkable anesthetic management process was stated. Therefore, we replaced these missing values with subgroup medians.

Table 1 gives an overview of basic demographic and treatment-related data, indicating no statistically significant difference over the four years in question with regard to ASA classification (I versus II), sex, age, or body weight. A significant difference did emerge for median time under anesthesia over the years, characterized by a distinct increase for the 2020 lockdown period (p = 0.049), even though the amount of time spent in the recovery room never significantly changed over the years (p = 0.28). Rates of intermediate care admission were again found to differ significantly (p = 0.01), but this finding was caused not by a deviation during the 2020 lockdown period but by a distinctly higher incidence of intermediate care admissions during the same season of 2017 (12.1%) compared to the years 2018 (3.5%), 2019 (4.7%), or even 2020 (4.8%).

**Table 1:** Pertinent basic data.

	2017		2018		2019		2020		
	n = 207		n = 231		n = 233		n = 125		
	n or ~x	(% or IQR)	n or ~x	(% or IQR)	n or ~x	(% or IQR)	n or ~x	(% or IQR)	p value
ASA 1 (n)	134	(64·7%)	163	(70·6%)	145	(62·2%)	89	(71·2%)	0·16*
ASA 2 (n)	73	(35·3%)	68	(29·4%)	88	(37·8%)	36	(28·8%)	
Female sex (n)	65	(31·0%)	96	(41·6%)	74	(31·8%)	43	(34·4%)	0·08*
Age (median months)	56	(12−144)	63	(16−133)	60	(10−122)	38	(11−113)	0·11†
Body weight (median kg)	17	(9−41)	19·6	(10−40)	19	(9−35)	15	(10−30)	0·29†
Time under anesthesia (median min)	58	(41−93)	57	(41−82)	54	(39−78)	64	(48−93)	0·049†
Time spent in recovery room (median min)	106	(83−165)	107	(81−155)	104	(72−146)	105	(73−168)	0·28†
Intermediate care admissions (n)	25	(12·1%)	8	(3·5%)	11	(4·7%)	6	(4·8%)	0·001*

Data are absolute numbers (n) with percentages (%) or median values (~x) with interquartile ranges (IQR).

*Pearson’s chi-squared test;

†Kruskal-Wallis test.

Overall, 37 events (4.7%) with a SpO_2_ below 90% were recorded.

Table 2 shows the types of surgery performed and the distribution over the study period.

**Table 2:** Types of surgery.

	2017		2018		2019		2020 = COVID lockdown time n = 125		Total		p value
	n = 207		n = 231		n = 233				n = 796		
Surgical procedures	n	%	n	%	n	%	n	%	n	%	0·322*
Abdominal wall reconstruction	1	(0·5%)	5	(2·2%)	2	(0·9%)	2	(1·6%)	10	(1·3%)	
Biopsy	7	(3·4%)	10	(4·3%)	8	(3·4%)	1	(0·8%)	26	(3·3%)	
Colorectal surgery	15	(7·2%)	7	(3·0%)	9	(3·9%)	10	(8·0%)	41	(5·2%)	
Diagnostics (CT/MRI)	0	(0·0%)	1	(0·4%)	3	(1·3%)	1	(0·8%)	5	(0·6%)	
Endoscopy: Bronchoscopy	6	(2·9%)	8	(3·5%)	3	(1·3%)	1	(0·8%)	18	(2·3%)	
Endoscopy: GI	37	(17·9%)	46	(19·9%)	45	(19·3%)	20	(16·0%)	148	(18·6%)	
Hernia surgery	47	(22·7%)	38	(16·5%)	39	(16·7%)	23	(18·4%)	147	(18·5%)	
Laparoscopic surgery	7	(3·4%)	6	(2·6%)	7	(3·0%)	7	(5·6%)	27	(3·4%)	
Laparotomy	1	(0·5%)	2	(0·9%)	1	(0·4%)	0	(0·0%)	4	(0·5%)	
Liver surgery	0	(0·0%)	1	(0·4%)	0	(0·0%)	0	(0·0%)	1	(0·1%)	
Others (minor procedures = e.g. dermoid cyst surgery, OK-432 instillation)	16	(7·7%)	31	(13·4%)	29	(12·4%)	18	(14·4%)	94	(11·8%)	
Splenectomy	1	(0·5%)	0	(0·0%)	0	(0·0%)	0	(0·0%)	1	(0·1%)	
Thoracic surgery	4	(1·9%)	3	(1·3%)	2	(0·9%)	1	(0·8%)	10	(1·3%)	
Tumor resection	2	(1·0%)	3	(1·3%)	1	(0·4%)	1	(0·8%)	7	(0·9%)	
Upper GI general surgery	7	(3·4%)	1	(0·4%)	3	(1·3%)	0	(0·0%)	11	(1·4%)	
Urology	50	(24·2%)	61	(26·4%)	76	(32·6%)	37	(29·6%)	224	(28·1%)	
Venous access (central): Implantation	6	(2·9%)	8	(3·5%)	5	(2·1%)	3	(2·4%)	22	(2·8%)	
Total	207	(100·0%)	231	(100·0%)	233	(100·0%)	125	(100·0%)	796	(100·0%)	

Data are absolute numbers (n) with percentages (%).

*Pearson’s chi-squared test.

Table 3 lists the results for the primary outcome (SpO_2_ events < 90%, lowest SpO_2_) stratified by patient age. None of them showed significant differences over the years, which applied to the overall results and to each age group, except for, albeit without a clinical correlate, for lowest heart rates among 13-to-18-year-olds.

**Table 3:** Primary outcome parameter.

	2017		2018		2019		2020		
	n or ~x	(% or IQR)	n or ~x	(% or IQR)	n or ~x	(% or IQR)	n or ~x	(% or IQR)	p value
All cases evaluated	n = 207		n = 231		n = 233		n = 125		
Events of SpO_2_ < 90% (n)	14	(6·8%)	9	(3·9%)	7	(3%)	7	(5·6%)	0·26*
Age 0−3 months	n = 19		n = 13		n = 13		n = 11		
Events of SpO_2_ < 90% (n)	2	(10·5%)	1	(7·7%)	0	(0·0%)	1	(9·1%)	0·71*
Age 4−12 months	n = 35		n = 32		n = 51		n = 24		
Events of SpO_2_ < 90% (n)	2	(5·7%)	4	(12·5%)	2	(3·9%)	2	(8·3%)	0·49*
Age 1−4 years	n = 44		n = 53		n = 43		n = 35		
Events of SpO_2_ < 90% (n)	2	(4·5%)	1	(1·9%)	2	(4·7%)	3	(8·6%)	0·54*
Age 5−12 years	n = 58		n = 83		n = 83		n = 35		
Events of SpO_2_ < 90% (n)	4	(6·9%)	1	(1·2%)	3	(3·6%)	1	(2·9%)	0·34*
Age 13−18 years	n = 51		n = 50		n = 43		n = 20		
Events of SpO_2_ < 90% (n)	4	(7·8%)	2	(4%)	0	(0%)	0	(0%)	0·18*

Data are as absolute numbers (n) with percentages (%) or median values (~x) with interquartile ranges (IQR).

*Pearson’s chi-squared test.

Table 4 summarizes the results for the various anesthesia techniques used, indicating a significant continuous decrease in secured airways matched by a continuous increase in natural-airway management over the years (p = 0.047). This finding reflects a steady development of regional anesthesia being increasingly performed under sedation only, thus being unrelated to the lockdown-specific preoperative management in early 2020.

**Table 4:** Anesthesia techniques.

	2017		2018		2019		2020		
	n = 207		n = 231		n = 233		n = 125		
	n	%	n	%	n	%	n	%	p value
Airway management									0·047*
Endotracheal intubation	75	(36·2%)	79	(34·2%)	65	(27·9%)	29	(23·2%)	
Laryngeal mask	43	(20·8%)	50	(21·6%)	48	(20·6%)	21	(16·8%)	
Natural airway with face mask	89	(43%)	102	(44·2%)	120	(51·5%)	75	(60%)	
Neuraxial or peripheral anesthesia									0·48*
Neuraxial regional	106	(51·2%)	101	(43·7%)	112	(48·1%)	52	(49·6%)	
Peripheral and trunk regional	14	(6·8%)	21	(9·1%)	12	(5·2%)	11	(8·8%)	

*Pearson’s chi-squared test.

Table 5 lists the rates of adverse events or complications related to anesthesia. Based on cases managed by general anesthesia, events were extremely rare (n = 3), and no events associated with regional anesthesia would be on record whatsoever had it not been for our inclusion of block failures (n = 4). None of these events associated with general or regional anesthesia highlighted any significant differences over the years, or, for that matter, between the period of ad-hoc preoperative management in early 2020 and the same calendar period of standard management over the preceding three years.

**Table 5:** Anesthesia-related adverse events.

	2017		2018		2019		2020		
	n = 207		n = 231		n = 233		n = 125		
	n	%	n	%	n	%	n	%	p value
Laryngospasm	0	(0%)	0	(0·9%)	0	(0%)	0	(0%)	‥
Bronchospasm	1	(0·5%)	2	(0·9%)	0	(0%)	0	(0%)	0·41*
Drug reaction	0	(0%)	0	(0%)	0	(0%)	0	(0%)	‥
Aspiration	0	(0%)	0	(0%)	0	(0%)	0	(0%)	‥
Cardiopulmonary resuscitation	0	(0%)	0	(0%)	0	(0%)	0	(0%)	‥
Intraoperative death	0	(0%)	0	(0%)	0	(0%)	0	(0%)	‥
Unexpected difficult airway	0	(0%)	1	(0·4%)	0	(0%)	0	(0%)	0·49*
High neuraxial block	0	(0%)	0	(0%)	0	(0%)	0	(0%)	‥
Spinal puncture during epidural anesthesia	0	(0%)	0	(0%)	0	(0%)	0	(0%)	‥
Failure of the neuraxial anesthetic technique	1	(0·9%)†	1	(1%)†	2	(1·8%)†	0	(0%)†	0·74*

*Pearson’s chi-squared test;

†percentage based on cases managed by neuraxial anesthesia.

Table 6 summarizes the findings from the logistic regression analysis for the surrogate marker of SpO_2_ dropping below the 90% threshold during intraoperative anesthesia management. Again, the ad-hoc strategy of preoperative management in early 2020 was not found to make a statistically significant difference to the incidence of these events (p = 0.367). The multivariate regression analysis did reveal that the likelihood of such events to occur was significantly increased in younger compared to older patients (p = 0.007), in cases of endotracheal intubation versus face-mask inhalation (p = 0.007), as well as in patients undergoing bronchopulmonary diagnostic and surgical procedures (p = 0.001).

**Table 6:** Logistic regression analysis for peripheral oxygen saturation (SpO_2_) drops <90%.

	Bivariate analysis			Multivariate analysis (fully adjusted)		
	Odds ratio (95% CI)		p value	Odds ratio (95% CI)		p value
Ad-hoc (first COVID-19 lockdown) versus standard pre-anesthetic management	1·26	(0·544−2·953)	0·583	1·49	(0·62−3·61)	0·367
Age (years)	0·95	(0·89−1·02)	0·175	0·90	(0·84−0·97)	0·007
Weight (kg)	0·99	(0·97−1·01)	0·582	not included	‥	‥
Female sex	0·99	(0·49−1·97)	0·978	not included	‥	‥
ASA classification (ASA I *vs* ASA II)	2·46	(1·27−4·79)	0·008	1·95	(0·97−3·91)	0·058
Bronchoscopy and lung surgery versus non-lung/non-thoracic procedure	6·48	(2·45−17·13)	<0·0001	7·20	(2·32−22·30)	0·001
Duration of procedure (per minute)	1·006	(1·001−1·011)	0·015	1·002	(0·99−1·008)	0·445
Endotracheal intubation *vs* face mask	2·58	(1·23−5·42)	0·012	3·36	(1·38−8·18)	0·007
Supraglottic airway *vs* face mask	1·19	(0·44−3·25)	0·722	1·08	(0·34−3·45)	0·891

ASA = American Society of Anesthesiologists; CI = confidence interval.

## Discussion

Confirming the study hypothesis, our findings demonstrate that an ad-hoc strategy of preoperative anesthesia management, as adopted by our center under the first COVID-19 lockdown in Austria, was not associated with an increase of the incidence of intraoperative respiratory or other anesthesia-related events in children and adolescents undergoing elective surgery.

At the time of writing, the pandemic still poses enormous challenges to perioperative medicine worldwide. Its first wave in early 2020 caught the medical community with no pertinent guidelines or standard operation procedures on hand. Swift implementation of unprecedented management strategies was required to maintain a basic level of surgical and anesthetic care [3, 4, 21, 22], and surgical procedures had to be cancelled in large numbers to keep intensive care resources in supply and protect medical staff [10, 23, 24]. Given the paramount role of social distancing in bringing down infection rates, the focus was on minimizing contacts between staff and patients.

Thus, the first Austrian lockdown imposed in late March of 2020 was characterized by social distancing requirements in the absence of specific guidelines on perioperative anesthesia management for elective surgical procedures. Moreover, no preoperative examinations of children were conducted as pediatric offices suspended their clinical routines to focus strictly on emergencies, and hospital staff was reduced by allocation to cohorts aimed at reducing intra-departmental transmission of virus. Against this background, the hospital administration gave its approval to implement a streamlined process of preoperative management as the only way to maintain a basic anesthetic and surgical service. The process was reduced to providing information on fasting requirements and admission dates by phone, without any face-to-face contact between patients, parents/legal guardians, and anesthetists until 15−30 minutes before anesthesia induction.

Respiratory complications are the most frequent critical events in pediatric anesthesia [25] in all age groups, given a 3.1% incidence reported across 261 European hospitals (APRICOT; Anesthesia Practice In Children Observational Trial [19]). The same study found a 1.9% incidence of hemodynamic instability, mainly in the wake of respiratory instability, in 61% of these events [19]. Yet the APRICOT cohort cannot be readily compared to the cases herein reported, given its 0.9% share of surgical procedures managed by regional anesthesia and sedation without airway instrumentation versus 48.5% in the present study. This large proportion is due to an ongoing strong focus we have been placing over the past two decades on developing and implementing specific techniques of regional anesthesia that allow spontaneous breathing of pediatric patients to be never interrupted [25–30].

Our clinical experience has been that this strategy of cutting down on invasive airway manipulation will lower the incidence of respiratory events, which, while not formally proving a causal relationship, is corroborated by the finding from our multivariate regression analysis that the risk for an SpO_2_ event < 90% was increased by a factor of 3.36 when endotracheal intubation was performed (see Table 6). Viewed in this way, our results do indicate that the reduction of airway manipulation may well have contributed to the very low incidence of respiratory events we noted, which included no perioperative events of hemodynamic instability whatsoever.

The opportunity for this study was incidental in that, outside of the circumstances of the first COVID-19 lockdown, no relevant data would have been available to conduct an analysis of modified *preoperative* management, and it would have been impossible to generate such data in the first place. It is, however, important to understand that we never modified our strategy of *intraoperative* and *postoperative* management in this situation. As stated above, an ever-growing proportion of anesthetic techniques conducted under mild sedation with a natural airway has been evolving at our center over many years [25–30] and certainly was not a result of special requirements associated with the pandemic, let alone an outcome of modifying our processes under its pressure. Such modifications, therefore, also cannot explain our secondary finding of significantly longer anesthesia durations during the lockdown in early 2020, which most likely was due to constraints in medical staff and a lack of highly experienced pediatric anesthetists during some of those days.

We realize the limitations of an observational retrospective study, one of them being different potential modifiers of respiratory risk during pediatric surgery like snoring or medication-related factors. Also, the incidence of respiratory adverse events is subject to seasonal variations [31], notably characterized by peaks of viral upper respiratory tract infections in children and adolescents during the winter. Last but not least, the limited case number we overlook within this ‘window of opportunity’ for study in early 2020 cannot definitively establish the true incidence of severe respiratory events if an ad-hoc strategy of preoperative management were to become the standard, also considering that the surgical case load of ASA I/II pediatric patients during this period in 2020 was down by 45.9% from the median case load of the period-matched previous three years.

All that being said, the findings for our primary endpoint (SpO_2_ events < 90%) are consistent with a safe preoperative management strategy, and our inclusion of elective ASA I/II cases only, while covering all common surgical procedures in children and adolescents, should make for a sample as representative as in other high-volume centers. Where our situation may differ from similar centers is in the high proportion of cases managed by regional anesthesia with mild sedation and an un-instrumented natural airway. This may have contributed to the very low incidence of respiratory events, given that most complications in pediatric anesthesia have a respiratory background [19].

A major reason for timely preoperative evaluation in pediatric anesthesia really is to anticipate the eventuality of difficult airways and of intraoperative emergencies due to co-morbidities [32]. Hence our findings suggest that an early process of patient assessment and obtaining informed consent may not by necessity improve the safety of pediatric anesthesia. Rather, it appears that the traditional approach of meeting face to face well in advance of the actual treatment is not so much a medical as a legal requirement. Regardless of the pandemic, therefore, it may be useful to reconsider applicable rules and regulations with a view to future strategies of pre-anesthetic management, which ideally should be explored in terms of telephone-based or ‘virtual reality’ environments.

## Supporting information

S1 Table Miscellaneous details of the study. (TIF)

S2 Table STROBE statement. Checklist of items that should be included in reports of observational studies according to STROBE.
(ZIP)
