# Supplementary figures and images for: Ad-hoc preoperative management and respiratory events in pediatric anesthesia during the first COVID-19 lockdown–an observational cohort study
Source: PLoS One. 2022 Aug 18;17(8):e0273353. doi: 10.1371/journal.pone.0273353 (PMC9387849; doi:10.1371/journal.pone.0273353)

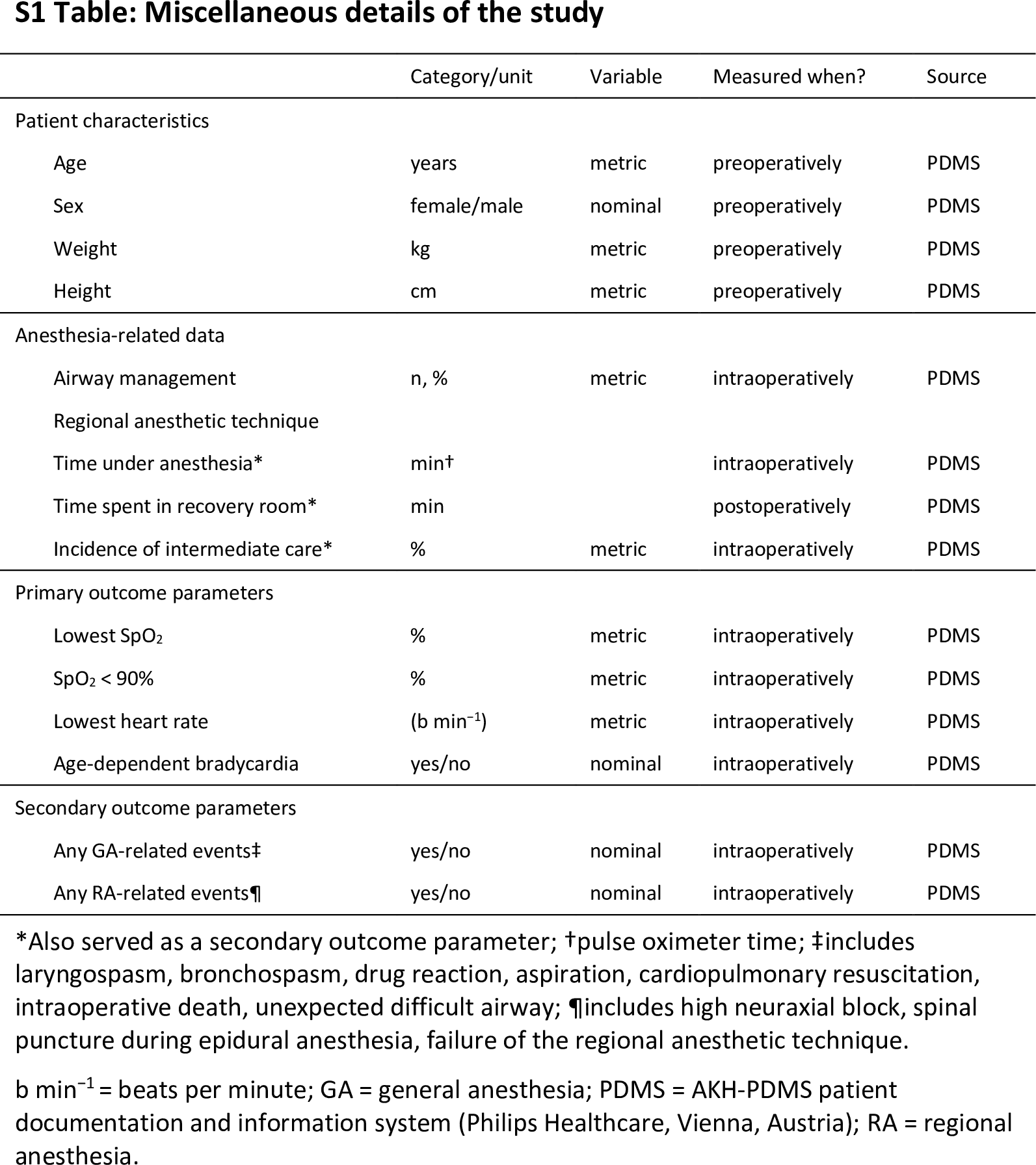

Supplement: S1 Table — (TIF) [file pone.0273353.s001.tif]

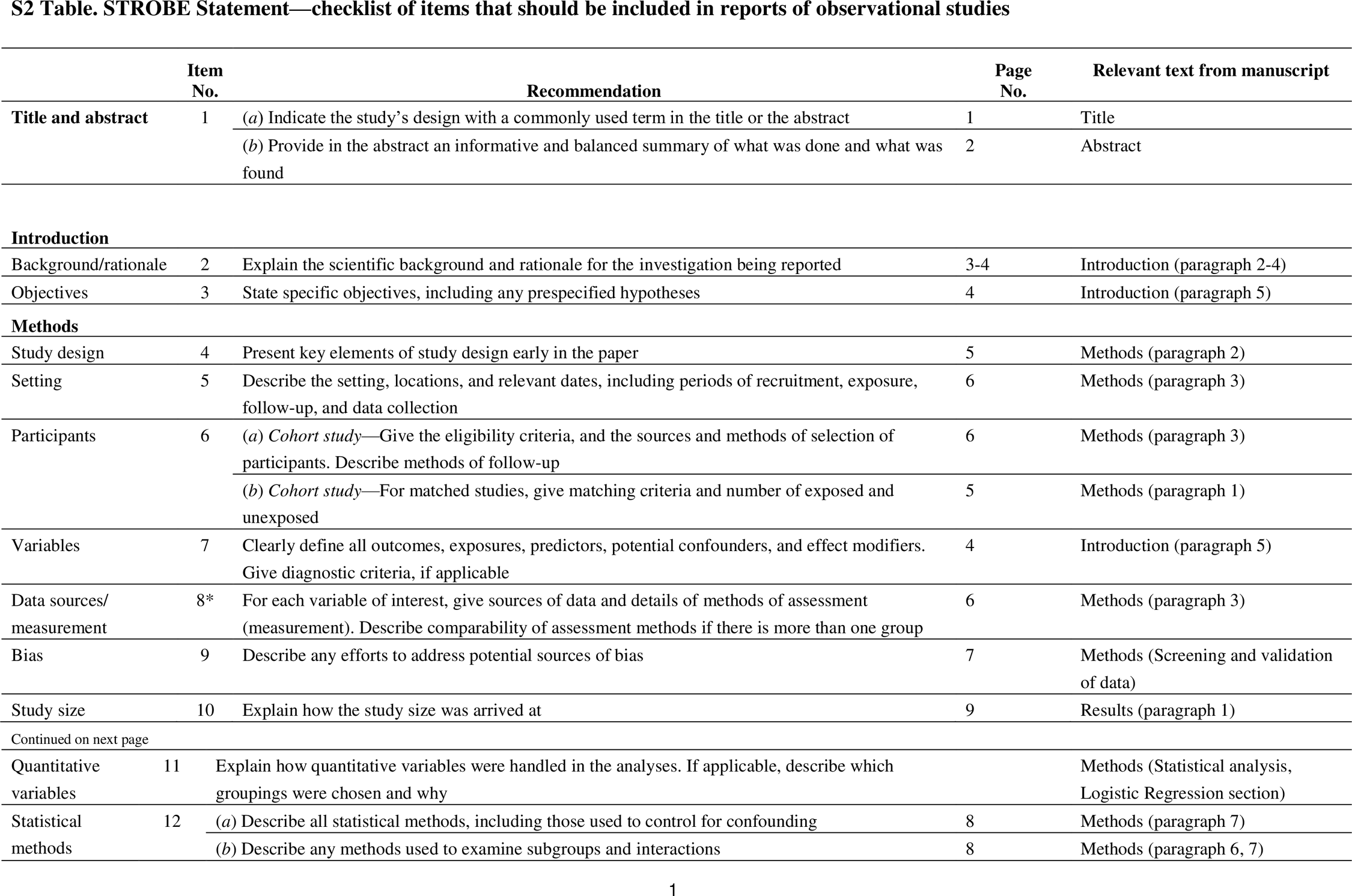

Supplement: S2 Table — Checklist of items that should be included in reports of observational studies according to STROBE. (ZIP) [file pone.0273353.s002.zip › S2_Table_1.tif]

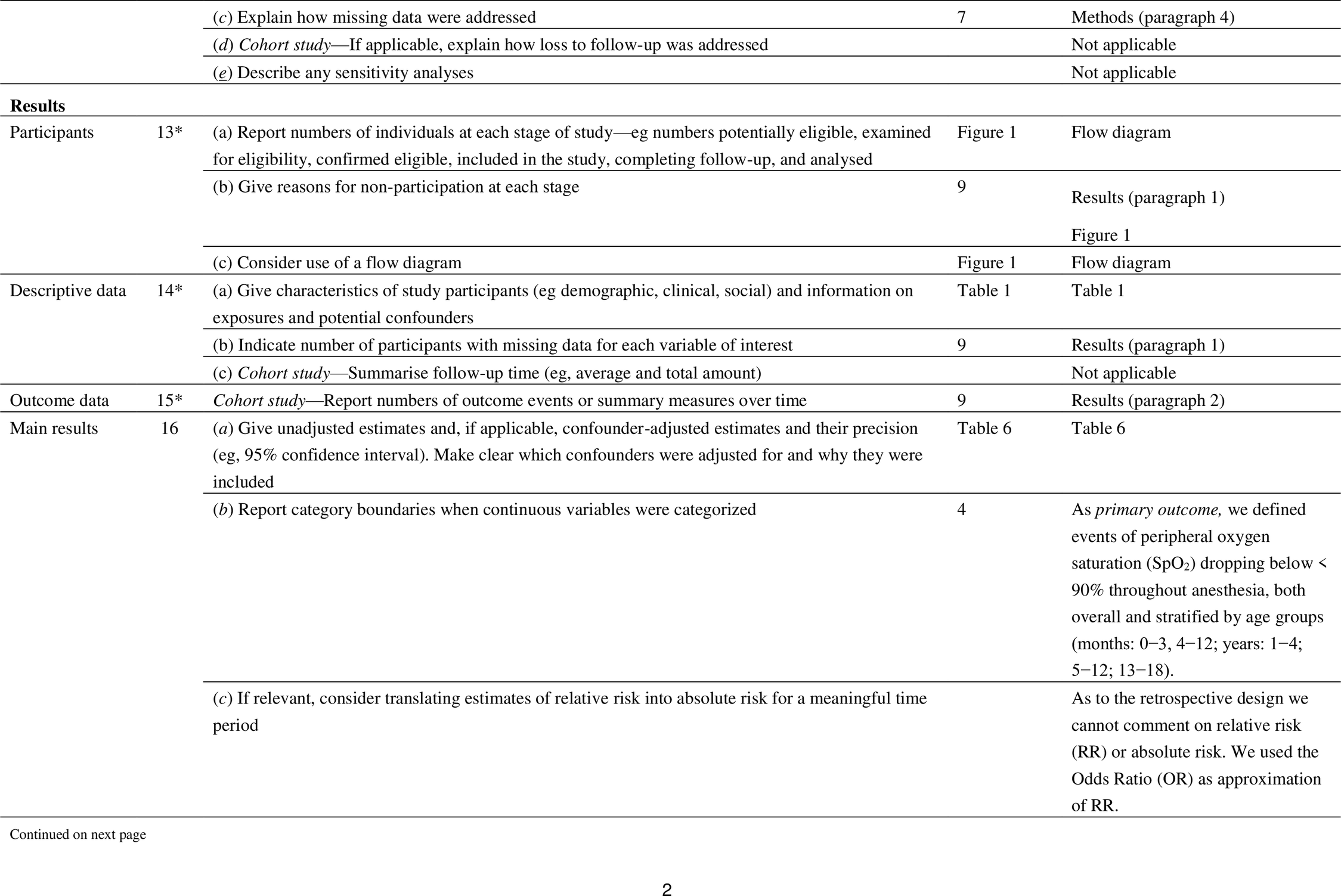

Supplement: S2 Table — Checklist of items that should be included in reports of observational studies according to STROBE. (ZIP) [file pone.0273353.s002.zip › S2_Table_2.tif]

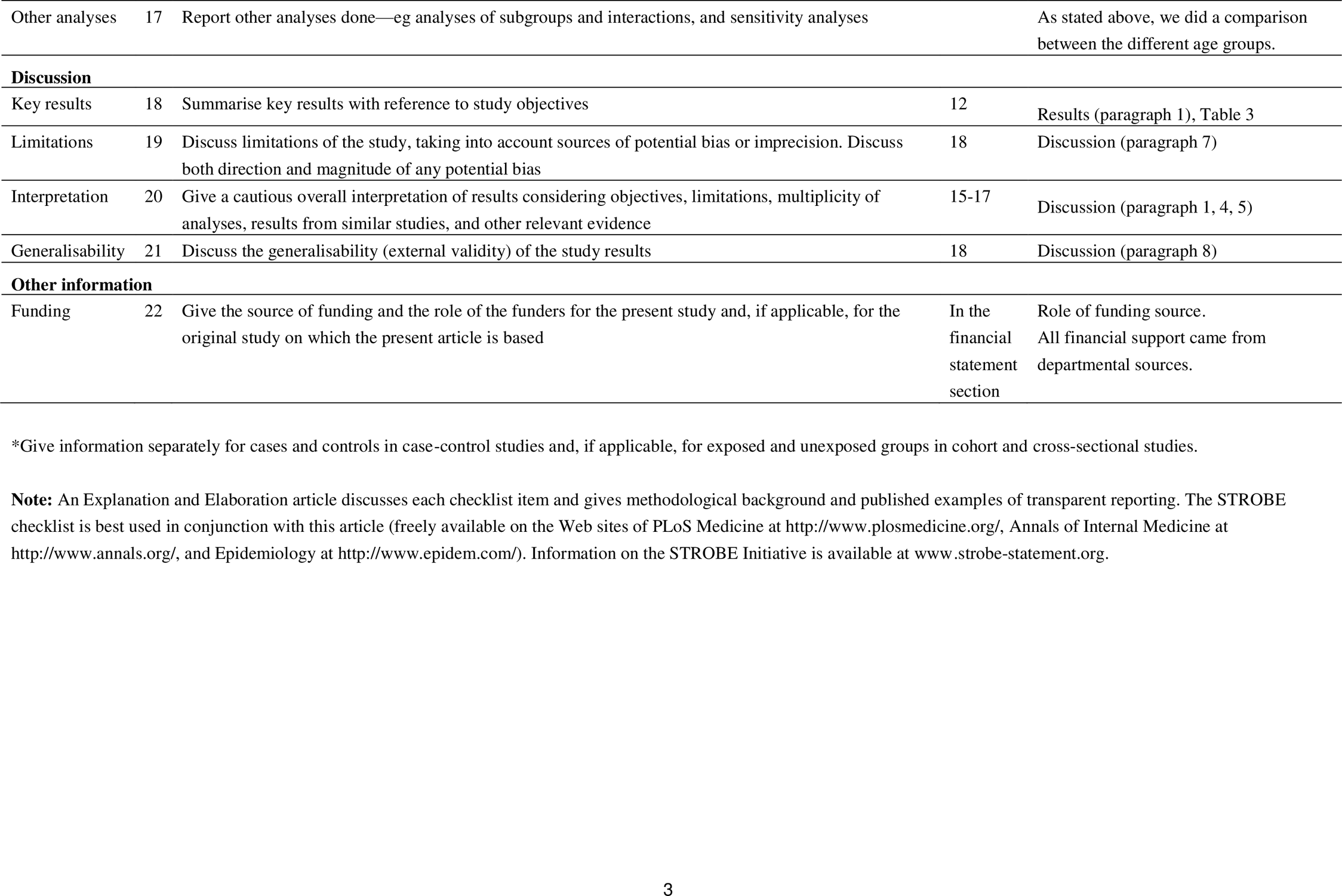

Supplement: S2 Table — Checklist of items that should be included in reports of observational studies according to STROBE. (ZIP) [file pone.0273353.s002.zip › S2_Table_3.tif]
